# Induction of cross-protection against influenza A virus by DNA prime-intranasal protein boost strategy based on nucleoprotein

**DOI:** 10.1186/1743-422X-9-286

**Published:** 2012-11-23

**Authors:** Jian Luo, Dan Zheng, Wenjie Zhang, Fang Fang, Hanzhong Wang, Ying Sun, Yahong Ding, Chengfei Xu, Quanjiao Chen, Hongbo Zhang, Ding Huang, Bing Sun, Ze Chen

**Affiliations:** 1 College of Life Sciences, Hunan Normal University, Changsha, Hunan 410081, China; 2 Shanghai Institute of Biological Products, Shanghai 200052, China; 3 State Key Laboratory of Virology, Wuhan Institute of Virology, Chinese Academy of Sciences, Wuhan, Hubei 430071, China; 4 Xinhua Hospital affiliated to Shanghai Jiaotong University of Medicine, Shanghai 200092, China; 5 State Key Laboratory of Bioreactor Engineering, East China University of Science and Technology, Shanghai 200237, China; 6 Institute Pasteur of Shanghai, Shanghai Institutes for Biological Sciences, Chinese Academy of Sciences, Shanghai 200025, China

**Keywords:** Influenza, Recombinant NP, DNA prime, Intranasal protein boost

## Abstract

**Background:**

The highly conserved nucleoprotein (NP) is an internal protein of influenza virus and is capable of inducing cross-protective immunity against different influenza A viruses, making it a main target of universal influenza vaccine. In current study, we characterized the immune response induced by DNA prime-intranasal protein boost strategy based on NP (A/PR/8/34, H1N1) in mouse model, and evaluated its protection ability against a lethal dose challenge of influenza virus.

**Results:**

The intranasal boost with recombinant NP (rNP) protein could effectively enhance the pre-immune response induced by the NP DNA vaccine in mice. Compared to the vaccination with NP DNA or rNP protein alone, the prime-boost strategy increased the level of NP specific serum antibody, enhanced the T cell immune response, and relatively induced more mucosal IgA antibody. The overall immune response induced by this heterologous prime-boost regimen was Th-1-biased. Furthermore, the immune response in mice induced by this strategy provided not only protection against the homologous virus but also cross-protection against a heterosubtypic H9N2 strain.

**Conclusions:**

The NP DNA prime-intranasal protein boost strategy may provide an effective strategy for universal influenza vaccine development.

## Background

Vaccination is the most safe and effective way to prevent influenza infection. The major mechanism of current influenza vaccine is based on induction of protective antibodies against the viral surface hemagglutin (HA) and neuraminidase (NA) which has been undergoing a high rate of mutation. Thus, to develop influenza vaccines that induce broad spectrum and robust immune response is a challenging task for researchers.

NP is a type-specific antigen which is highly conserved. The amino acid sequence similarity of NP is above 90% within same type of influenza virus [1]. Furthermore, NP is the major antigen recognized by cytotoxic T cell (CTL) after viral infection. NP-specific CTLs can promote lysis of infected cells by recognizing the NP peptide-MHC complex presented by the virus-infected cells. Thus, they contribute to the clearance of the virus from the infected tissue and prevent the spread of viral infection. Those CTLs which are able to induce cross-reaction against NP play an important role in control of viral infection [2,3]. Therefore, induction of strong NP-specific immune response, particularly cell mediated immunity, is an aim for developing universal vaccine. Currently, various forms of universal influenza vaccines targeting NP have been reported in animal models. These vaccines include recombinant protein vaccines based on eukaryotic or prokaryotic expression system, vaccines based on viral or bacterial carrier, and DNA vaccines, among which DNA vaccine is the most investigated [4-9]. However, results from several groups and our previous study indicated that immunization with NP DNA vaccine alone was insufficient to induce well heterosubtypic immunity [10-13]. Additionally, although some exciting progresses have been made in small animal tests, the immunogenicity of DNA vaccine in large animals, quadrumana, and human is still limited and further optimization is needed on vaccine design, delivery system, and immunization strategy, etc.

The respiratory tract mucosa is the site of influenza viral infection and the local immune responses on mucosal surfaces play an important role in defense of viral infection. Therefore, mucosal immunity against influenza virus has received much attention in recent years. A study by Nguyen *et al.* has demonstrated that the acquisition of heterosubtypic protective immunity was relevant to CTL response in local mucosal lymphoid tissue [14]. In a study on heterosubtypic immunity response induced by DNA prime-adenoviral vector boost strategy based on NP and Matrix protein-2 (M2), Price *et al.* revealed that compared to intramuscular injection, intranasal administration of adenovirus vector vaccine in mice and ferrets induced not only higher systemic immune response, but also stronger and more durable mucosal immunity with effective protection against heterosubtypic virus [15,16]. Moreover, several research teams including our group have successfully induced cross-protective immunity against influenza virus by using inactivated vaccine and recombinant NP, Matrix protein-1(M1) and M2 vaccines with mucosal adjuvants [5,17-19]. In this study, highly conserved internal NP was selected as a target antigen and a DNA prime-intranasal protein boost strategy was adopted to immunize mice. We confirmed that the NP DNA prime-intranasal protein boost was able to induce systemic and local mucosal immune responses, which could effectively provide a cross-protection against homologous and heterosubtypic influenza virus.

## Results

### Protection against lethal PR8 virus challenge in mice by DNA prime-intranasal protein boost strategy based on NP

Plasmids pCAGGSP7/NP and rNP were prepared as described in our previous study [5,11]. The expression of the cloned NP gene was confirmed by Western blot analysis [11]. The purified rNP was also confirmed by SDS-PAGE and Western blotting analysis [5].

One hundred and fourteen mice were randomized into 6 groups, with 19 mice in each group. Mice were immunized as described in the section of methods. Briefly, group D1 received one dose of 100 μg NP DNA vaccine; group P1 received one dose of 50 μg rNP vaccine; group D2 received two doses of 100 μg NP DNA vaccine; group D1P1 received one dose of 100 μg NP DNA vaccine followed by one dose of 50 μg rNP; group D2P1 received two doses of NP DNA vaccine followed by one dose of rNP vaccine. As for the immunization, the DNA vaccine was administrated by *in vivo* electroporation and rNP was intranasally (i.n) administrated under anesthesia. The interval between immunizations was 2 weeks and the control group was unimmunized. All mice were i.n. challenged with a lethal dose (5 × LD_50_) of A/PR/8/34 (H1N1) viral suspension 3 weeks post-immunization. On day 3, 5 and 7 after the lethal challenge, 3 mice from each group were randomly sacrificed. The bronchoalveolar wash was collected and used for virus titration. The survival rates and the body weight losses of the rest 10 mice in each group were monitored for 21 days after the challenge to evaluate the protection effect against A/PR/8/34 (H1N1) virus.

**Figure 1:**
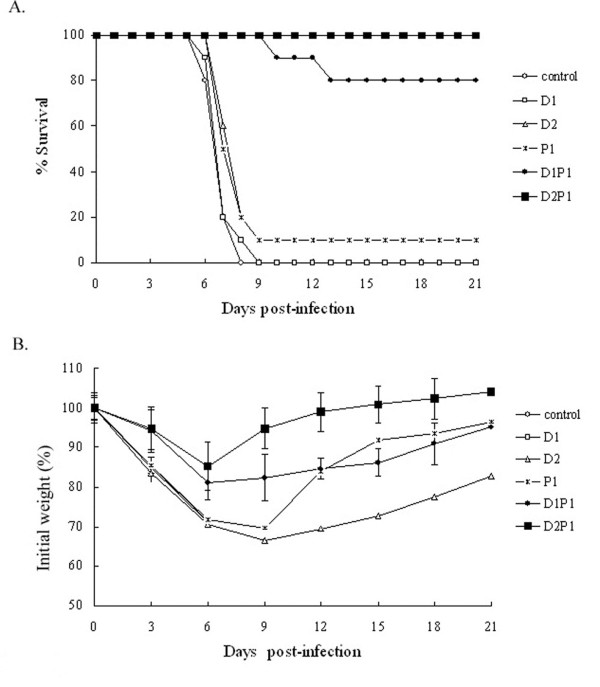
**Survival rates (A) and body weight changes (B) after challenge with lethal homologous influenza virus.** One hundred and fourteen mice were randomly divided into six groups. Group D1 received one dose of 100 μg NP DNA vaccine; group P1 received one dose of 50 μg rNP vaccine; group D2 received two doses of 100 μg NP DNA vaccine; group D1P1 received one dose of 100 μg NP DNA vaccine followed by one dose of 50 μg rNP; group D2P1 received two doses of NP DNA vaccine followed by one dose of rNP vaccine. 3 weeks after the last immunization, mice were challenged with a lethal dose (5×LD_50_) of influenza PR8 virus. Survival rates (**A**) and body weight loss (**B**) were monitored for 21 days.

The results present in Table 1 showed that the control group and the group immunized with one dose of NP DNA vaccine alone failed to provide any protection, and the body weight of mice continued to decline and all mice died within 9 days after the challenge (Figure 1A). Although survival rates of 10% were observed in the group receiving two doses of NP DNA vaccine and the group receiving rNP alone, there were no significant differences compared with that of the control group. The body weight losses of these two groups were similar with that of control group. However, in groups immunized with once or twice NP DNA vaccine followed by an intranasal boost with rNP (Group D1P1 and D2P1), the body weight of mice decreased to a mild extent compared to that of previously described groups and recovered very soon (Figure 1B). Mice in these two groups were well protected and the protection rates were 80% and 100%, respectively. Although two mice died in Group D1P1, the date of death was delayed to day 13 after the challenge (Figure 1A). These results suggest that the NP DNA prime-intranasal protein boost strategy is capable of providing mice with protective immunity against the lethal dose challenge of homologous influenza virus.

**Table 1:** Protection against lethal PR8 virus challenge in mice by DNA prime intranasal protein boost strategy based on NP

**Group**	**Immunization**		**Lung virus titer (log**_**10**_**TCID**_**50**_**/ml)**^**a**^			**Survival rate (No. of survivors/no. tested)**
	**DNA prime**	**rNP boost**	**3 days**	**5 days**	**7 days**	
D1	once	-	7.24±0.25	6.39±0.08	5.02±0.45	0/10
D2	twice	-	7.14±0.43	5.63±0.32	4.35±0.14 ^b^	1/10
P1	-	once	6.94±0.42	5.78±0.42	5.24±0.25	1/10
D1P1	once	once	6.41±0.16^b^	4.68±0.39^b^	1.16±0.29^b^	8/10^b^
D2P1	twice	once	6.13±0.43^b^	4.89±0.44^b^	0.33±0.58^b^	10/10^b^
Control	-	-	7.19±0.17	5.89±0.16	5.28±0.25	0/10

Mice were immunized as described in section of methods. 3 weeks after the last immunization, mice were challenged with a lethal dose (5×LD_50_) of influenza PR8 virus. Bronchoalveolar washes from three mice in each group were collected 3 days, 5 days and 7 days post-infection for titration of lung virus respectively. The survival rate of mice 21 days post-infection was determined.

^a^ Results are expressed as mean ± SD of tested mice in each group.

^b^ Displays significant difference compared with mouse in control groups (P<0.05).

The changes in lung virus titers in mice on day 3, 5 and 7 after the challenge were shown in Table 1. The lung virus titer of each group was at a high level (more than 6 log_10_TCID_50_/ml) on day 3 after challenge. There were no significant differences between Group D1, D2, P1 and the control group (P>0.05), whereas the titers in Group D1P1 and D2P1 were significantly lower as compared to that of the control group (P<0.05). Five days after the challenge, the lung virus titers in Group D1P1 and D2P1 decreased greatly while only small decreases occurred in other groups. By day 7 post-infection, there was almost no detectable virus in Group D2P1 (below 1.0 log_10_TCID_50_/ml) and only tiny amount (about 1.2 log_10_TCID_50_/ml) in Group D1P1. The lung virus titers in other groups remained at a relatively high level (more than 4.0 log_10_TCID_50_/ml) despite some drops. In conclusion, although the DNA prime-intranasal protein boost strategy could not prevent mice from viral infection, this strategy was able to promptly remove the virus, reduce the lung viral load, and mediate effective protection.

### Serum and mucosal antibody responses in mice induced by DNA prime-intranasal protein boost strategy based on NP

Forty-eight mice were randomized into 6 groups, with 8 mice in each group. Concentrations of NP-specific IgG in serum, IgA in nasal and bronchoalveolar wash were detected at week 3 after the last immunization. As shown in Table 2, all groups except the control had a clear serum antibody response, among which the antibody responses induced by NP DNA vaccine were enhanced significantly by a dose of intranasal protein boost in Group D1P1 and D2P1 (P<0.05). Furthermore, antibody titer was markedly improved by the two doses of DNA vaccination as compared to a single dose DNA vaccination before the protein boost (P<0.05). In case of none DNA preimmunization, one dose of i.n. administration of rNP alone produced relatively lower antibody titer. These results indicated that the heterologous NP DNA prime-intranasal protein boost strategy was able to enhance the serum antibody response induced by NP DNA vaccine. Further analysis on serum antibody isotypes revealed that vaccination with DNA vaccine alone, either once or twice, induced mainly IgG2a antibody, while the antibody induced by i.n. administration of rNP alone was dominated by IgG1. On the other side, both IgG2a and IgG1 antibody levels were obviously improved by the DNA prime-intranasal protein boost strategy, as compared to the single component vaccination method. However, IgG2a was still the major composition of the antibody isotypes, indicating that the intranasal protein boost did not change the bias of antibody isotype induced by DNA vaccine.

**Table 2:** Serum and mucosal antibody responses in mice induced by DNA prime-intranasal proteinboost strategy based on NP

**Group**	**Immunization**		**Ab responses (ELISA, 2**^**n**^**)**^**a**^			**Serum IgG subclasses (ELISA, 2**^**n**^**)**^**a**^		**IgG2a/ IgG1 Ratio**
	**DNA prime**	**rNP boost**	**Serum IgG**	**Nasal wash IgA**	**Lung wash IgA**	**IgG2a**	**IgG1**	
D1	once	-	11.40±2.17	-	-	11.83±2.71	10.25±0.96	2.99
D2	twice	-	17.00±0.70	-	-	18.44±1.23	16.10±0.79	5.06
P1	-	once	18.20±0.45	0.89±0.19	6.50±0.53	15.50±1.37	17.89±1.01	0.19
D1P1	once	once	21.60±0.89^b, c^	2.67±0.46^c^	8.67±0.87^c^	21.55±1.50	19.25±0.82	4.92
D2P1	twice	once	24.80±0.83^c, d, e^	4.56±0.38^c, e^	11.30±1.12^c, e^	24.11±1.00	20.80±0.92	9.92
Control	-	-	-	-	-	-	-	-

Mice were immunized as described in section of methods. 3 weeks after the last immunization, serum, nasal wash, and lung wash specimens of five mice in each group were prepared and examined by ELISA for NP-specific IgG, IgA, IgA Abs respectively.

^a^ Results are expressed as mean ± SD of five tested mice in each group.

^b^ Displays significant difference compared with mouse in D1group (P<0.05).

^c^ Displays significant difference compared with mouse in P1 group (P<0.05).

^d^ Displays significant difference compared with mouse in D2 group (P<0.05).

^e^ Displays significant differences compared with mouse in D1P1 group (P<0.05).

We also evaluated the IgA levels in the nasal wash and bronchoalveolar wash from each group. As shown in Table 2, a single dose of rNP intranasal alone produced only low level of mucosal IgA in mice, but higher titer of IgA antibody was detected at the mucous membrane of the respiratory tract in prime-boost groups (Group D1P1 and D2P1, P<0.05). It was also found that more IgA was induced by increasing the frequency of DNA vaccination. These results show that DNA prime-intranasal protein boost strategy is therefore effective in inducing local mucosal antibody response.

### Cellular immune responses in mice induced by DNA prime-intranasal protein boost strategy based on NP

**Figure 2:**
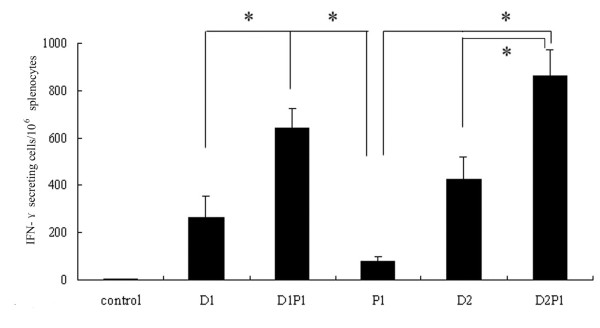
**The numbers of INF-γ secreting splenic CD8**^**+**^**T cells in vaccinated mice.** Mice were immunized as described in method section. 3 weeks after the last immunization, the number of IFN-γ secreting CD8^+^ T cells in the spleen from the different groups of mice was evaluated by ELISpot. The results represent the averages of triplicate wells of three mice, and are expressed as means ± SD, * Significant difference (P<0.05).

ELISpot was conducted to detect the cellular immune response induced by the DNA prime-intranasal protein boost strategy based on NP in three mice of each group. 3 weeks after the last immunization, splenocytes were isolated from mice and stimulated with MHC-I epitope peptide from NP *in vitro*. The amount of cells secreting IFN-γ was measured after the stimulation. As shown in Figure 2, considerable amounts of IFN-γ secreting CD8^+^ T cells were induced by NP DNA vaccination alone, either once or twice. On the contrary, single i.n. administration of rNP alone (Group P1) induced only tiny amount of IFN-γ secreting CD8^+^ T cells. An intranasal boost with rNP after once or twice DNA vaccinations could effectively increase the number of IFN-γ secreting CD8^+^ T cells (P<0.05). The results from ELISpot illustrated that the DNA prime-intranasal protein boost strategy based on NP could induce more IFN-γ secreting CD8^+^ T cells in mice. In other words, this prime-boost immunization strategy effectively enhanced the CD8^+^ T cellular immune response induced by DNA preimmunization.

**Figure 3:**
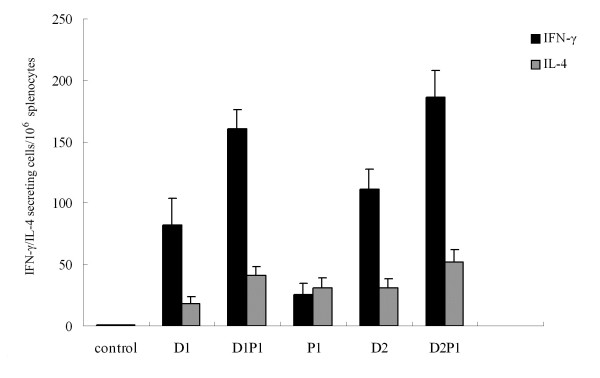
**The numbers of IFN-γ / IL-4 secreting splenic CD4**^**+**^**T cells in vaccinated mice.** Mice were immunized as described in method section. 3 weeks after the last immunization, the number of IFN-γ /IL-4 secreting CD4^+^ T cells in the spleen from the different groups of mice was evaluated by ELISpot. The results represent the averages of triplicate wells of three mice, and are expressed as means ± SD.

To determine the response of CD4^+^ T cells and the differentiation trend of Th cells in each immunization group, splenocytes from mice were stimulated with NP MHC-II epitope peptides (see section of methods) and the number of IFN-γ or IL-4 secreting cells post-stimulation was measured. From the results in Figure 3, it was clear that the numbers of IL-4 and especially IFN-γ secreting CD4^+^ T cells were significantly increased by the intranasal protein boost after the DNA vaccination (P<0.05), as compared to DNA or intranasal protein vaccination alone, proving that this prime-boost strategy was efficient in enhancing the CD4^+^ T cell immune response. In addition, the numbers of IFN-γ and IL-4 secreting CD4^+^ T cells from the same group were compared. In groups vaccinated with DNA vaccine alone and with DNA prime-intranasal protein boost strategy, the number of CD4^+^ T cells secreting IFN-γ was significantly higher than that secreting IL-4 (P<0.05), indicating that the immune responses induced were Th-1-biased. Only in group immunized with intranasal rNP alone, the IFN-γ secreting CD4^+^ T cells were less than IL-4 secreting CD4^+^ T cells, revealing a Th-2-biased response. These results showed that the DNA prime-intranasal protein boost strategy based on NP could induce well Th-1-biased cellular immune response.

### Heterologous protection against lethal H9N2 avian influenza virus in mice by DNA prime-intranasal protein boost strategy based on NP

**Figure 4:**
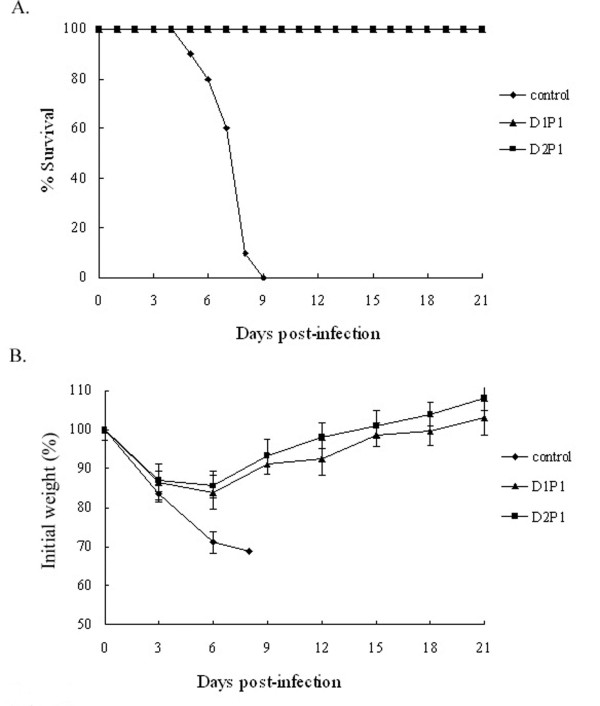
**Survival rates (A) and body weight changes (B) after challenge with lethal heterologous influenza virus.** Mice were immunized as described in method section. 3 weeks after the last immunization, mice were challenged with a lethal dose (5×LD_50_) of influenza A/Chicken/JiangSu/07/2002(H9N2). Survival rate (**A**) and body weight loss (**B**) were monitored for 21 days.

To evaluate the efficacy of the NP DNA prime-intranasal protein boost strategy against lethal dose challenge of heterologous virus, 57 mice were randomized into 3 groups with 19 mice in each group. Two groups received one or two doses of NP DNA vaccine followed by an intranasal boost with rNP at an interval of 2 weeks and the rest group was unimmunized for control. All mice were i.n. challenged at week 3 after the last immunization with 5 × LD_50_ of A/Chicken/JiangSu/07/2002 (H9N2). Three mice from each group were randomly taken for residual lung virus titer on day 3, 5 and 7 after the challenge. The rest 10 mice in each group were monitored for 21 days after the challenge, and their survival rates and body weight losses were recorded. By day 3 post-infection, obvious signs of influenza infection occurred in all groups of mice. With the symptom increased, mice in the control group began to die since day 5 and all died by day 9 post-infection. However, the symptom relieved in the two immunization groups around day 7 after the infection, resulting in 100% protection rates (Figure 4A). For the body weight of mice after the challenge, weight losses occurred in all groups. Compared to the control group, mice in two immunized groups lost less weight and started to recover by day 6 post-infection, and reached normal state around day 20 post-infection (Figure 4B). In conclusion, the NP DNA prime-intranasal protein boost strategy could provide protective immunity for mice against lethal challenge of heterologous influenza virus.

We monitored the lung virus titer of mice on day 3, 5 and 7 after the lethal dose challenge of heterologous influenza virus A/Chicken/JiangSu/07/2002 (H9N2). The results are shown in Table 3. It was observed that either one dose or two doses of NP DNA vaccine followed by an intranasal boost with rNP could not prevent viral infection. Although both lung virus titers of mice from the two immunized groups reached high levels by day 3 after the challenge, they were still lower than that of the unimmunized group (P<0.05). By day 5 post-infection, the lung virus titers of the two immunized groups declined remarkably as compared to that of the unimmunized group (P<0.05). Lung viruses were almost cleared in mice of the two immunized groups and only extremely low viral levels were detected (below 1.0 log_10_TCID_50_/ml) by day 7 post-infection. However, the lung virus titer in control group remained at a relatively high level, which was more than 6.0 log_10_TCID_50_/ml. These results indicated that the DNA prime-intranasal protein boost strategy based on NP could accelerate the clearance of lung virus after the infection with heterologous virus, reduce the lung virus load, and achieve the protective effect.

**Table 3:** Protection of mice against lethal heterologous influenza A virus challenge by DNA prime-intranasal protein boost strategy based on NP vaccine

**Group**	**Immunization**		**Lung virus titer (log**_**10**_**TCID**_**50**_**/ml)**^**a**^			**Survival rate (No. Of survivors/no. tested)**
			**3 days**	**5 days**	**7 days**	
	**DNA prime**	**rNP boost**				
D1P1	once	once	6.41±0.13^b^	4.61±0.35^b^	0.67±0.58^b^	10/10^b^
D2P1	twice	once	6.22±0.16^b^	4.00±0.33^b^	0.41±0.71^b^	10/10^b^
Control	-	-	7.17±0.24	6.72±0.25	6.17±0.58	0/10

Mice of three groups were immunized as described above. 3 weeks after the last immunization, mice were challenged with a lethal dose (5×LD_50_) of influenza virus A/Chicken/JiangSu/07/2002 (H9N2). Bronchoalveolar washes from three mice in each group were collected 3 days, 5 days and 7 days post-infection for titration of lung virus respectively.

The survival rate of mice 21 days post-infection was determined.

^a^ Results are expressed as mean ± SD of tested mice in each group.

^b^ Displays significant difference compared with mouse in control groups (P<0.05).

## Discussion

The high conservation and known effect in protective immunity against influenza of NP make it a high potential target antigen for universal influenza vaccine. Extensive studies have been carried out on influenza vaccine targeting NP for a long time, among which DNA vaccine is being the most investigated. Results from our previous study indicated that NP DNA vaccine alone could achieve better protection only in the case of repeated immunization [11,12,20]. Currently, heterologous prime-boost strategies have become the primary way to enhance the immunogenicity of the DNA vaccine. Immunization strategy based on DNA prime followed by a viral vector or recombinant protein boost has been widely applied in mouse model, nonhuman primate, and human clinical trials [21,22]. Epstein *et al.* have induced cross-reactive CTL by using DNA prime-adenovirus vector vaccine boost strategy based on NP and protected mice from lethal challenge of H5N1 virus [10]. So far, we developed NP DNA prime-intranasal protein boost strategy, and evaluated the induced immune response and protective effect in mouse model.

Our results demonstrated that NP DNA immunization followed by intranasal boosting with rNP could effectively enhance the humoral immune response by producing higher titer of NP specific antibody in serum, as compared to vaccination with either NP DNA vaccine or intranasal rNP alone. Classification and bias of antibody isotypes can somehow reflect the Th bias of induced immune response. It is generally believed in mouse model that it is a Th-1-biased immune response when the ratio IgG2a/IgG1 of serum antibody isotype titers is larger than 1, whereas IgG2a/IgG1<1 indicates a Th-2-biased immune response [23]. In our experiments, the Th-1-biased immune response induced by NP DNA vaccine was maintained following an intranasal boosting with rNP and the response level was raised as compared to vaccination with DNA or rNP alone. These results are consistent with those of other research groups [24-26].

Several studies have demonstrated that NP-specific antibody is not directly related to protective immunity against influenza virus. Our previous serum passive immunization experiment also proved that NP-specific serum alone could not provide protection [5,27]. However, there were studies which revealed that non-neutralizing antibodies against NP might play a role in fighting against a sublethal dose challenge of viral infection [28,29]. Recently, two studies conducted by Lamere *et al.* suggested that NP-specific serum antibody could play a certain role in heterosubtypic immunity against influenza virus through mechanisms with both FcRs and CD8^+^ T cells involved [30,31]. Although NP-specific serum antibody is not the key factor in protective immunity, it should be taken into account in vaccine design concerning its potential protective mechanism. In this study we showed that the heterologous prime-boost strategy could induce good antibody responses.

Specific cellular immune responses targeting influenza internal conservative antigen (i.e. NP) are widely regarded as the main factor mediating cross-protection against influenza protection [32,33]. NP-specific CTLs can rapidly proliferate, differentiate, and be recruited to infection sites (lung tissue and nasal mucosa) after viral infection. These CTLs kill viral infected cells through direct killing, FasL dependent or TRAIL dependent pathway and mediate clearance of virus [34,35]. On the other hand, Th-1-type CD4^+^ T cells can mediate protective immunity through IFN-γ dependent or nondependent mechanism [36,37]. Vaccine that targeting conversed gene and immunizing through mucosal route with a valid immune strategy is one of the effective methods to induce heterosubtypic immunity against influenza. Recently, our group successfully induced heterosubtypic immunity against influenza by intranasal administration of NP, M1 and M2 protein with mucosal adjuvant [5,18,19].

In our experiments, we induced high levels of NP-specific IFN-γ–producing CD8^+^ T cells and Th-1-biased CD4^+^ T cells, through a DNA prime-intranasal protein boost strategy, which was significantly different from vaccination with DNA or protein alone. In addition, the strength of such immune was actually found to be associated with the survival rate of mice after the homo- or heterologous viral challenge. Moreover, from the lung virus titer between day 3 and day 7 post-infection, it was clear that virus in lung was cleared from mice in intranasal protein boost group while there were no big changes in group vaccinated with DNA or protein alone. These results suggest that the NP DNA prime-intranasal protein boost strategy can induce higher level of NP-specific T cellular immune response, particularly CD8^+^ T cell response, significantly augmenting the protection efficiency of NP based DNA vaccine.

The intranasal protein boost strategy also induced high level of NP specific mucosal IgA antibody during the experiments. Meanwhile, well protection was obtained in the two mucosal boost groups (Group D1P1 and D2P1), indicating somehow that the NP-specific mucosal IgA was related to protection against influenza infection. This finding was consistent with our previous results [5]. Although the role of NP-specific IgA in protection against influenza infection remained unclear, an *in vivo* experiment conducted by Mukhtar *et al.*[38] may provide some possible explanations. It was proved in their work that single-chain intracellular antibody against NP could specifically bind to newly synthesized NP in cell after influenza infection and block the interaction between NP and influenza RNA polymerase complexes. By this way, the transcription and translation of influenza viral gene were depressed. Additionally, several studies indicated that the binding of IgA to its poly-receptor on epithelial cells enabled IgA to bind the newly synthesized viral protein during its penetration through epithelial cells and thus, influenced the viral replication [39-41]. Therefore we speculate that NP specific IgA may interfere with the viral replication to somewhat extent during its secretion in respiratory epithelium.

NP is the target to induce influenza heterosubtypic immunity. We observed in our studies that NP DNA vaccination once or twice followed by an intranasal boost with rNP was able to protect mice from lethal challenge of heterologous H9N2 virus. This type of infection-permissive heterosubtypic immunity accelerated the clearance of lung virus after infection obviously and the protection rate reached 100%. It was reported previously that in the cross-protective immunity mediated by NP specific CD8^+^ T cells, ideal effect was achieved only when vaccine antigen and the NP of the challenge strain shared the identical sequences of the immunodominant protective CTL epitopes [42]. Here we found the homology of the amino acid sequence was 93.6% between NP of PR8 (H1N1) strain and the Chicken/Jiangsu/11/2002 (H9N2) strain. They had the same immunodominant CTL epitope-NP_147-155_(TYQRTRALV) and differences only occurred in 1-2 amino acids of the other 3 immunodominant Th epitopes. For this reason, CTL specific to NP of PR8 virus is also able to kill H9N2 viral infected cells and mediate cross-protection. Since less is known about the B cell epitope on NP and its conservative property [43], the role of NP-specific antibodies (IgG and sIgA) in cross-protection is not clear yet. The more broad-spectrum cross-protection induced by DNA prime-intranasal protein boost strategy based on NP, such as cross-protection against highly pathogenic avian influenza H5N1 virus and pandemic H1N1 2009 influenza A virus, will be further evaluated in our future study.

In conclusion, our study proved the DNA prime-intranasal protein boost strategy based on NP could effectively enhance the immune response induced by NP DNA vaccine. Meanwhile, this vaccine delivery via the mucosal route was able to induce better mucosal immune response. Immune response induced by this strategy was not only resistant to lethal challenge of homologous influenza virus, but also provided complete cross-protection. Due to its success in inducing heterosubtypic immunity against influenza virus, the DNA prime-protein intranasal boost strategy based on NP will probably be a good choice for universal flu vaccine research

## Methods

### Viruses and mice

Influenza viruses used in this study included a mouse adapted A/PR/8/34 (H1N1) virus and an H9N2 influenza virus A/Chicken/Jiangsu/7/2002 (H9N2), which were obtained through lung-to-lung passages and adapted in mice as described in our previous studies [44]. They were stored at -70 ºC until use. Specific-pathogen-free (SPF) female BALB/c mice (6–8 weeks old) were purchased from Shanghai Laboratory Animal Center, China. All mice were bred in the animal resource center at Shanghai Institute of Biological Products and maintained under specific-pathogen-free conditions. All experiments involving animals were approved by the Animal Care Committee of Shanghai Institute of Biological Products.

### DNA plasmids and recombinant nucleoprotein

Plasmids pCAGGSP7/NP was constructed by cloning the PCR products of NP gene from the A/PR/8/34 (H1N1) influenza virus strain into the plasmid expression vector pCAGGSP7, as described in our previous study [11]. The plasmid was propagated in *E. coli* XL1-blue bacteria and purified using QIAGEN purification kits (QIAGEN-tip 500). DNA was resuspended in sterile physiological saline at a concentration of 1 mg/ml and stored at −20°C.

rNP was produced in *E. coli* as described in our previous study [5]. Briefly, *E. coli* BL21 (DE3) bacteria was transformed by using the recombinant plasmid pET28a/NP containing NP gene from the A/PR/8/34(H1N1) influenza virus strain. Bacteria grew in a manner of log phase and protein expression was induced by adding isopropyl-β -D- thiogalactopy -ranoside to a final concentration of 0.1 mM. After 6 h of further incubation at 28°C, the cells were pelleted, resuspended and lysated. The soluble His-tagged rNP in cell lysate supernatant was then purified by affinity chromatography using a nickel-charged Sepharose affinity column (QIAGEN) according to the manufacturer’s instructions. The rNP were dialyzed against PBS and sterile filtered, stored in a final concentration of 2 mg/ml at –80°C for later use.

### Immunization and challenge

Six groups of SPF female BALB/c mice, 6-8 weeks of age, were vaccinated by one dose of NP DNA vaccine (D1 group), two doses of NP DNA vaccine (D2 group), one dose of rNP (P1 group), one or two doses of NP DNA followed by one dose of rNP (D1P1 or D2P1 group) respectively, the unimmunized group served as negative control. For DNA vaccine immunization, 50 μl pCAGGSP7/NP plasmid were applied to both quadriceps femoris muscles. Immunization was followed immediately by electroporation of the injected area [45]. For protein immunization, mice were anesthetized and immunized intranasally with 20 μl of PBS containing one dose of 50 μg rNP. The time interval between immunizations is 2 weeks. 3 weeks after the last immunization, mice were anesthetized and challenged intranasally with 20 μl of the viral suspension containing 5×LD_50_ of A/PR/8/34 (H1N1) or 5×LD_50_ of A/Chicken/Jiangsu/7/2002 (H9N2). Survival and body weight loss were monitored for 21 days.

### Specimens

3 weeks after the last immunization, five mice from each group were taken for sample collection. The mice were anaesthetized with chloroform and then bled from the heart with a syringe. The sera were collected from the blood and used for IgG Ab assays. After bleeding, the mice were incised ventrally along the median line from the xiphoid process to the point of the chin. The trachea and lungs were taken out and washed three times by injecting with a total of 2 ml PBS containing 0.1% BSA. The head of the mouse was removed and the lower jaw was cut off. A syringe needle was inserted into the posterior opening of the nasopharynx and then a total of 1 ml PBS containing 0.1% BSA was injected three times to collect the outflow as nasal wash. The bronchoalveolar and nasal wash was centrifuged to remove cellular debris and used for IgA Ab assays. Bronchoalveolar washes from three mice in each group were also collected 3 days, 5 days and 7 days post-infection for titration of lung virus respectively [46].

### ELISA

The concentration of IgG and IgA against rNP was measured by ELISA. ELISA was performed using a series of reagents consisting of: first, 2 μg/ml rNP; second, serial 2-fold dilutions of sera or nasal wash or bronchoalveolar wash from each group of mice; third, goat anti-mouse IgG Ab (γ-chain specific) (KPL) or goat anti-mouse IgA(α-chain specific) (KPL) conjugated with horseradish peroxidase (HRP); and finally, the substrate 3,3',5,5'- Tetramethylbenzidine (TMB). The amount of chromogen produced was measured based on absorbance at 450 nm. Ab-positive cut-off values were set as means +2×SD of unimmunized sera. An ELISA Ab titer was expressed as the highest serum dilution giving a positive reaction.

### ELISpot assays

Spleen cells were isolated from mice for IFN-γ/IL-4 ELISpot assays at 3 weeks after the last immunization and processed as described in our previous study [12]. The cell suspensions were stimulated in the presence of 4 μg /ml NP peptide stimulants for 24 h at 37°C. The choice of peptides used in this assay was based on previous reports [47,48]. We used the H-2d-restricted NP class I peptide (TYQRTRALV) and a pool of three H-2^d^-restricted class II peptides (FWRGENGRKTRSAYERMCNILKGK, RLIQNSLTIERMVLSAFDERNK, and AVKGVGTMVMELIRMIKRGINDRN). Spots were counted with an ELISpot reader system (Bioreader 4000; Bio-sys, Germany). The number of peptide-reactive cells was represented as spot forming cells per 10^6^ splenocytes and was calculated by subtracting spot numbers in control peptide (HIV pol peptide ILKEPVHGV) wells from that in NP specific peptide-containing wells [49]. For IL-4 ELISpot assays, a pool of three H-2d-restricted class II peptides were used as stimulants.

### Virus titration

The bronchoalveolar wash was diluted by 10-fold serially starting from a dilution of 1:10, inoculated to MDCK cells at 37°C and examined for cytopathic effect 3 days later. The virus titer of each specimen, expressed as the 50% tissue culture infection dose (TCID_50_), was calculated by the Reed-Muench method. The virus titer in each experimental group was represented by the mean ± SD of the virus titer per ml of specimens from three mice in each group [46].

### Statistics

The results of test groups were analyzed statistically by Student’s t-test; if *P*-value is less than 0.05, the difference was considered significant. The survival rates of the mice in test and control groups were compared by using Fisher’s exact test.

## Abbreviations

Ab: Antibody; BSA: Bovine serum albumin; ELISA: Enzyme-linked immunosorbent assay; ELISpot: Enzyme-linked immunospot; CTL: Cytotoxic T cell; LD_50_: 50% lethal dose; NP: Nucleoprotein; M1: Matrix protein-1; M2: Matrix protein-2; PBS: Phosphate buffered saline; SPF: Sspecific pathogen free; TCID_50_: 50% tissue culture infection dose.

## Competing interests

The authors declare that they have no conflict of interests.

## Authors' contributions

JL carried out most of the experiments. DZ, FF and YS were responsible for part of the experiment. CFX participated in manuscript preparation. YHD, QJC, HBZ and DH participated in antibody detection and lung virus titration. HZW and BS participated in its design and coordination. ZC and WJZ were the main designers of the experiment and prepared the manuscript. All authors read and approved the final manuscript.
